# Supplementary material for: Catalytic Pyrolysis of PET Polymer Using Nonisothermal Thermogravimetric Analysis Data: Kinetics and Artificial Neural Networks Studies
Source: Polymers (Basel). 2022 Dec 24;15(1):70. doi: 10.3390/polym15010070 (PMC9824759; doi:10.3390/polym15010070)
Supplement: Supplementary file 1 [file polymers-15-00070-s001.zip › polymers-2115900-supplementary.pdf]

Table S1
Kinetic parameters obtained by Coats-Redfern model

Reaction Mechanis	Heating Rates												Average		
	2 K/min			5 K/min			10 K/min			20 K/min					
	Ea kJ/mol	**ln A**	R^2	Ea kJ/mol	**ln A**	R^2	Ea kJ/mol	**ln A**	R^2	Ea kJ/mol	**ln A**	R^2	Ea kJ/mol	**ln A**	R^2
F1	146	23.29	0.9996	179	30.1	0.9991	139	22.47	0.9995	242	40.53	0.9999	177	29	0.9995
F2	258	44.26	0.9944	272	47.62	0.9974	176	29.33	0.9975	281	47.55	0.9986	247	40	0.9970
F3	402	70.9	0.9872	386	68.82	0.9956	218	37.08	0.9947	324	55.27	0.9957	333	54	0.9933
D1	155	24.07	0.9884	230	38.56	0.9999	228	37.13	0.9999	426	71.13	0.9993	260	39	0.9969
D2	193	30.61	0.9944	269	45.26	0.9999	247	39.99	1	448	74.36	0.9997	289	43	0.9985
D3	246	38.95	0.9986	317	52.84	0.9996	268	42.45	0.9999	472	77.09	1	326	48	0.9995
D4	210	32.33	0.9963	285	46.76	0.9998	254	39.81	1	456	74.27	0.9998	301	44	0.9990
A2	67	10.8	0.9995	84	12.64	0.999	64	13.56	0.9994	115	18.61	0.9999	83	16	0.9995
A3	41	14.95	0.9993	52	14.16	0.9989	39	17.32	0.9993	73	13.06	0.9999	51	16	0.9994
A4	28	16.9	0.9993	36	16.59	0.9987	26	19.04	0.9992	52	16.25	0.9999	36	18	0.9993
R1	72	10.51	0.9864	109	16.94	0.9999	108	16.54	0.9999	207	34.21	0.9992	124	21	0.9964
R2	104	14.63	0.9969	141	22.39	0.9997	123	18.7	1	224	36.59	0.9999	148	24	0.9991
R3	117	16.69	0.9984	153	24.25	0.9996	128	19.29	0.9999	230	37.25	1	157	25	0.9995
P2	30	16.79	0.9807	49	14.88	0.9998	48	16.11	0.9998	98	15.37	0.9991	56	17	0.9949
P3	16	18.54	0.9707	29	17.84	0.9998	28	18.88	0.9998	61	14.89	0.999	34	18	0.9923
P4	9	19.16	0.9509	19	19.15	0.9997	18	20.09	0.9997	43	17.6	0.9988	22	20	0.9873

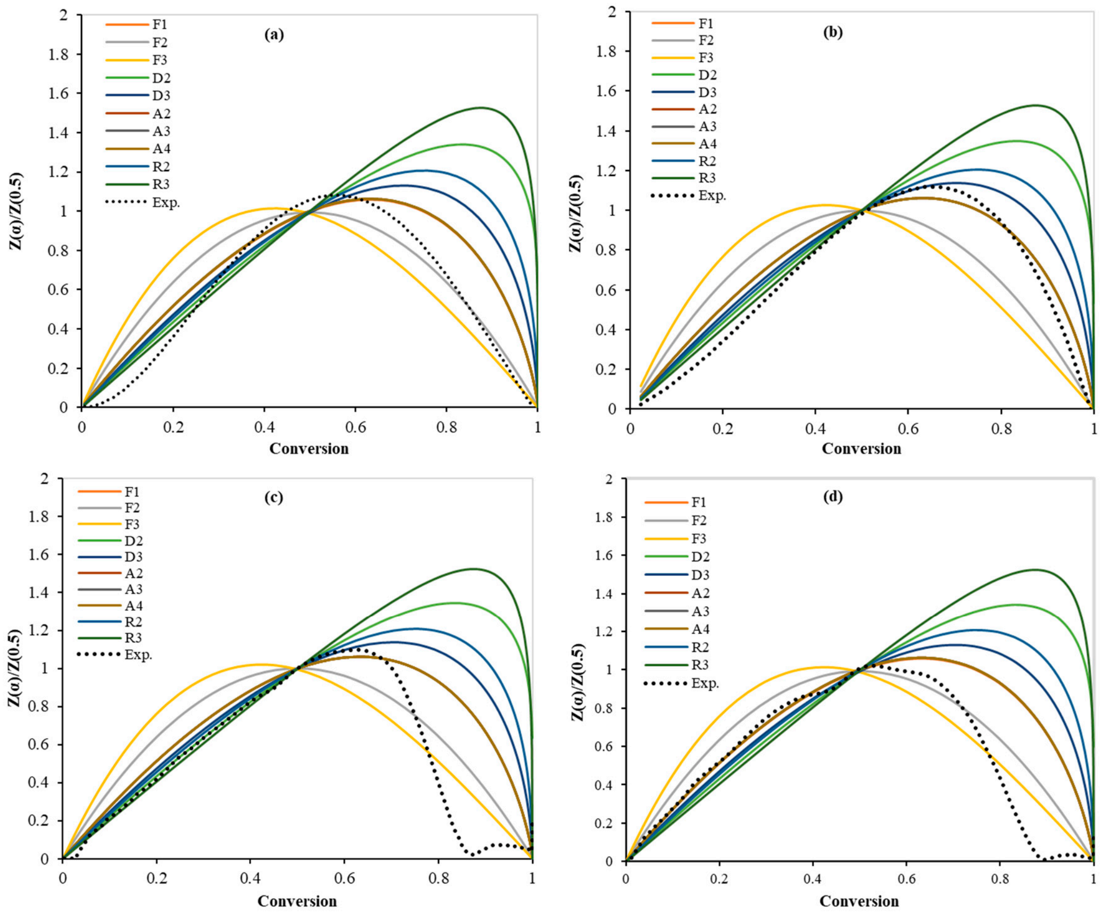

Figure S1. Criado model master plots of catalytic cracking of PET at different heating rate: (a) 2 K/min, (b) 5 K/min, (c) 10 K/min, and (d) 20 K/min.
